# Losartan Ameliorates Calcium Oxalate-Induced Elevation of Stone-Related Proteins in Renal Tubular Cells by Inhibiting NADPH Oxidase and Oxidative Stress

**DOI:** 10.1155/2018/1271864

**Published:** 2018-04-24

**Authors:** Baolong Qin, Qing Wang, Yuchao Lu, Cong Li, Henglong Hu, Jiaqiao Zhang, Yufeng Wang, Jianning Zhu, Yunpeng Zhu, Yang Xun, Shaogang Wang

**Affiliations:** Department of Urology, Tongji Hospital, Tongji Medical College, Huazhong University of Science and Technology, Wuhan, China

## Abstract

Calcium oxalate (CaOx) is the most common type of urinary stone. Increase of ROS and NADPH oxidase gives rise to inflammation and injury of renal tubular cells, which promotes CaOx stone formation. Recent studies have revealed that the renin-angiotensin system might play a role in kidney crystallization and ROS production. Here, we investigated the involvement of Ang II/AT1R and losartan in CaOx stone formation. NRK-52E cells were incubated with CaOx crystals, and glyoxylic acid-induced hyperoxaluric rats were treated with losartan. Oxidative stress statuses were evaluated by detection of ROS, oxidative products (8-OHdG and MDA), and antioxidant enzymes (SOD and CAT). Expression of NADPH oxidase subunits (Nox2 and Nox4), NF-*κ*B pathway subunits (p50 and p65), and stone-related proteins such as OPN, CD44, and MCP-1 was determined by Western blotting. The results revealed upregulation of Ang II/AT1R by CaOx treatment. CaOx-induced ROS and stone-related protein upregulation were mediated by the Ang II/AT1R signaling pathway. Losartan ameliorated renal tubular cell expression of stone-related proteins and renal crystallization by inhibiting NADPH oxidase and oxidative stress. We conclude that losartan might be a promising preventive and therapeutic candidate for hyperoxaluria nephrolithiasis.

## 1. Introduction

Calcium oxalate (CaOx) is the major constituent of most urinary stones, and hyperoxaluria is one of the primary risk factors for idiopathic kidney stone [1]. Renal tubular cells are notably injured in states of high levels of oxalate or CaOx crystals, which is associated with the development of oxidative stress (OS) and overproduction of reactive oxygen species (ROS) [2]. Intracellular ROS are generated mainly by nicotinamide adenine dinucleotide phosphate (NADPH) oxidase and mitochondria and involved in a variety of signaling pathways.

NADPH oxidase has emerged as a major source of receptor-mediated ROS production during exposure to high levels of oxalate or CaOx crystals [3, 4]. Activation of NADPH oxidase increases generation of ROS that modulate the expression of several stone-related macromolecules that promote or inhibit the formation of kidney stones [5]. The NADPH oxidase inhibitor apocynin has been proven to inhibit crystal deposition by regulating oxidative stress levels and stone-related protein expression [6]. Osteopontin (OPN), monocyte chemotactic protein 1 (MCP-1), and CD44 are very common stone-related proteins in urolithiasis research.

Recent studies have revealed that the renin-angiotensin system (RAS) might play an important role in kidney crystallization and ROS production in hyperoxaluric rats [7]. Angiotensin II (Ang II) type 1 receptor (AT1R) inhibitors were able to reduce renal injury and crystal deposition [8]. However, the mechanism underlying Ang II/AT1R in CaOx stone formation remains unclear. This study was designed to investigate Ang II/AT1R involvement in CaOx-induced activation of NADPH oxidase and expression of subsequent stone-related proteins and to identify new promising drug targets for prevention and treatment of CaOx stones.

## 2. Materials and Methods

### 2.1. Reagents

Calcium oxalate monohydrate (COM) crystals were purchased from Macklin (Shanghai, China). COM crystals were weighed and suspended in sterile phosphate buffer solution at 1 mM and then were diluted into different concentrations. Ang II was purchased from Sangon Biotech (Shanghai, China). Anti-AT1R, anti-NADPH oxidase 2 (Nox2), anti-p50, and anti-p65 antibodies were purchased from Proteintech (Wuhan, China). Losartan and apocynin were purchased from Sigma Aldrich (St. Louis, MO, USA). 2′,7′-Dichlorofluorescein diacetate (DCF-DA) and Fluo-3AM were purchased from Beyotime Institute of Biotechnology (Shanghai, China). Anti-NADPH oxidase 4 (Nox4), anti-OPN, and anti-MCP-1 antibodies were purchased from Abcam (Cambridge, MA, USA). An anti-CD44 antibody was purchased from Cell Signaling Technology (Boston, MA, USA). Malondialdehyde (MDA), superoxide dismutase (SOD), and catalase (CAT) assay kits were purchased from Nanjing Jiancheng Bioengineering Institute (Jiangsu, China). An 8-OHdG assay kit was purchased from Elabscience (Wuhan, China). The transfection reagent Lipofectamine™ 2000 was purchased from Invitrogen (Carlsbad, CA, USA).

### 2.2. Animal Model

Eight-week-old male Sprague-Dawley rats (200–220 g) were purchased from the Experimental Animal Center of Tongji Hospital, Tongji Medical College, Huazhong University of Science and Technology. All animal experiments were performed according to the Guide for the Care and Use of Laboratory Animals (published by National Academy Press, Washington, DC, 2011) and were approved by the Ethical Committee of Tongji Hospital (permit number: TJ-A20151202). Hyperoxaluria was induced by daily intraperitoneal injections of glyoxylic acid (80 mg/kg) for 2 weeks. Angiotensin type 1 receptor blocker losartan (80 mg/kg) dissolved in saline was administrated by gavage for 2 weeks simultaneously. Four groups of eight rats each were used in the study: group A, untreated control animals; group B, hyperoxaluria induction only; group C, hyperoxaluria with losartan treatment; and group D, losartan treatment only.

### 2.3. Cell Culture and Treatments

The normal rat kidney proximal tubular epithelial cell line, NRK-52E, was obtained from the Type Culture Collection of the Chinese Academy of Sciences (Shanghai, China). Cells were maintained in Dulbecco's modified Eagle's medium (DMEM) (Hyclone; USA) supplemented with 10% fetal bovine serum (FBS) (Gibco; Grand Island, NY, USA) at 37°C in a humidified atmosphere with 5% CO_2_. NRK-52E cells were stimulated with COM at a series of concentrations (0, 0.1, 0.5, 1, 5, or 10 mM) for 3–48 h. Cells were pretreated with NADPH oxidase inhibitor apocynin (30 *μ*M) or AT1R inhibitor losartan (10 *μ*M) for 1 h and then exposed to COM.

### 2.4. Small Interfering RNA (siRNA) Transfection

Three different siRNAs targeting specific sequences of the AT1R gene and a negative control siRNA were designed and synthesized by GenePharma Co. Ltd. (Shanghai, China). Sequences of siRNAs are shown in Table 1. NRK-52E cells were seeded in 24-well plates (5 × 10^4^ cells/well) and cultured in DMEM containing 10% FBS until 60%–70% confluence. After starvation for 6 hours, the siRNA transfection was performed according to manufacturer's instructions. The culture medium was replaced with serum-free DMEM 6 hours after transfection. Then, COM or Ang II (1 *μ*M) were added to stimulate the cells for subsequent assays.

### 2.5. Detection of Intracellular ROS by Flow Cytometry

Intracellular ROS production was detected using the probe DCF-DA. Cells were seeded in a 6-well plate and stimulated by COM and other drugs as aforementioned, followed by incubation with 10 *μ*M DCF-DA for 30 min at 37°C. Then, cells were collected and washed with PBS. After resuspending the pellet in 200 *μ*l PBS, fluorescence was detected by a flow cytometer (BD Biosciences).

### 2.6. Measurement of Oxidative and Antioxidative Biomarkers

MDA was measured as a product of lipid peroxidation, and 8-OHdG was regarded as a marker of oxidative DNA damage. SOD and CAT activities as well as MDA content were determined by chemiluminescence methods. The concentration of 8-OHdG considered as a marker of oxidative DNA damage was measured using a commercial ELISA kit according to the manufacturer's instructions.

### 2.7. Quantitative Polymerase Chain Reaction (PCR)

Total RNA was extracted from kidney tissues and NRK-52E cells using TRIzol reagent (Invitrogen). Total cDNA was synthesized using the PrimeScript® RT reagent kit (Takara Biotechnology, Dalian, China). Real-time PCR was performed using an ABI Prism 7500 system (Applied Biosystems, Foster City, CA, USA) according to manufacturer's instructions. Sequences of primers are as follows: AT1R, forward CACCATCTGCATAGCGTATT and reverse TTCGTAGACAGGCTTGAGTG; *β*-actin, forward CACGATGGAGGGGCCGGACTCATC and reverse TAAAGACCTCTATGCCAACACAGT. PCR thermal cycling conditions were as follows: denaturation at 95°C for 30s, followed by 40 cycles of 95°C for 30 s and 60°C for 30 s. Relative changes of gene expression were calculated using 2^−ΔΔ*Ct*^ and expressed as fold changes relative to the control group.

### 2.8. Western Blotting Analysis

Kidney tissues and NRK-52E cells were lysed with RIPA buffer containing 1% protease inhibitor PMSF. The supernatant was gathered after centrifugation. All lysates were diluted with SDS loading buffer and boiled for 5 minutes. Equal amounts of protein (40 *μ*g) were loaded into each lane of a 10% SDS-PAGE gel for electrophoresis. Proteins were then transferred onto a PVDF membrane. Then, the membranes were blocked with 5% BSA for 2 h and incubated with primary rabbit polyclonal antibodies against AT1R (1 : 1000), Nox2 (1 : 1000), Nox4 (1 : 2000), p50 (1 : 1000), p65 (1 : 2000), OPN (1 : 1000), MCP-1 (1 : 2000), or GAPDH (1 : 1000) or a mouse polyclonal antibody against CD44 (1 : 1000) at 4°C overnight, followed by incubation with a secondary antibody (anti-rabbit or anti-mouse IgG) for 1 h at 37°C. Membranes were developed using an enhanced chemiluminescence Bio-Rad Clarity Western ECL kit. Band intensities were analyzed using ImageJ software. Protein levels were normalized to GAPDH expression.

### 2.9. Morphological Staining

Paraffin-embedded kidney tissue sections were subjected to Von Kossa staining to identify CaOx crystal depositions, according to manufacturer's instructions.

### 2.10. Statistical Analysis

Results are presented as the mean ± SEM. One-way ANOVA was used to test significant differences among groups with SPSS version 18.0. A value of *P* < 0.05 was considered as statistically significant.

## 3. Results

### 3.1. Effects of Various Concentrations of Ang II on ROS Generation in NRK-52E Cells

**Figure 1:**
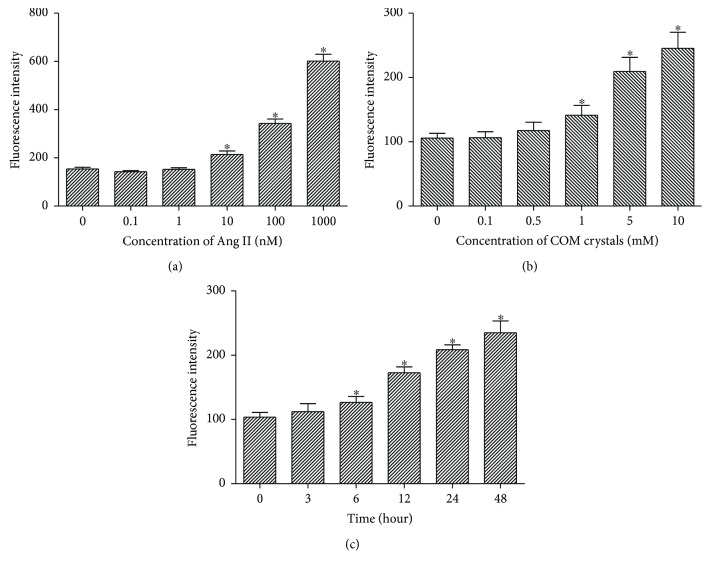
Effects of Ang II and COM on ROS generation in NRK-52E cells. (a) Cells were treated with various Ang II (0.1, 1, 10, 100, and 1000 nM) for 6 h, and then intracellular ROS was detected by flow cytometry. (b) Cells were treated with various COM (0, 0.1, 0.5, 1, 5, and 10 mM) for 6 h, and then intracellular ROS was detected by flow cytometry. (c) Cells were treated with COM (1 mM) for 0, 3, 6, 12, 24, and 48 h, and then intracellular ROS was detected by flow cytometry. The data are expressed as mean ± SEM. ^∗^*P* < 0.05 compared with the control group.

Confluent NRK-52E cells were treated with Ang II at a series of concentrations (0.1, 1, 10, 100, and 1000 nM) for 6 h to determine the effect of Ang II on intracellular ROS generation. Treatment with 1 nM Ang II or lower did not influence ROS generation (Figure 1(a)). Therefore, 1 nM Ang II was added to the medium as a supplement in subsequent experiments to simulate the physiological state.

### 3.2. Effects of COM on ROS Generation in NRK-52E Cells Are Concentration- and Time-Dependent

NRK-52E cells were exposed to various COM concentrations (0, 0.1, 0.5, 1, 5, and 10 mM) for various periods of time (0, 3, 6, 12, 24, and 48 h) to identify the appropriate concentration and time for treatment. ROS generation in response to COM was both concentration- and time-dependent (Figures 1(b) and 1(c)). And exposure to 1 mM COM for 6 h was adopted as the appropriate treatment condition.

### 3.3. Ang II/AT1R Expression Was Increased during Exposure to CaOx Crystals *In Vitro* and *In Vivo*

**Figure 2:**
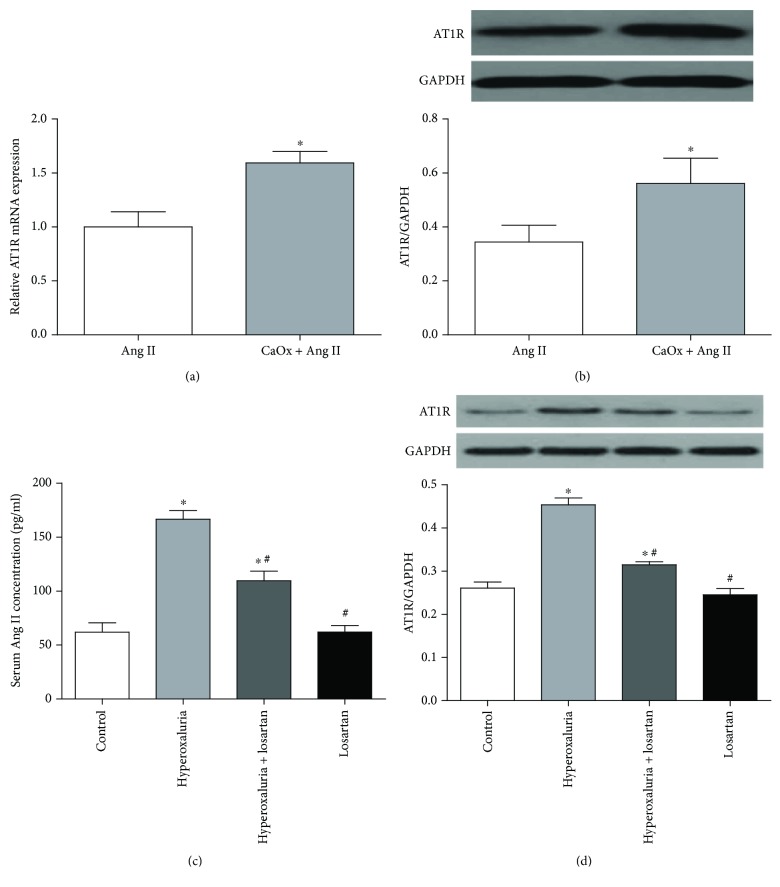
Ang II/AT1R expression was increased during exposure to CaOx crystals which was alleviated by losartan. NRK-52E cells were cultivated with COM (1 mM) for 6 h; the expression of AT1R was increased at both mRNA (a) and protein (b) levels. ^∗^*P* < 0.05 compared with the Ang II group. (c) The serum concentration of Ang II was increased in the hyperoxaluria group compared with the untreated control group which was alleviated by losartan administration. (d) AT1R expression of the hyperoxaluria group was upregulated compared with the untreated control group which was also alleviated by losartan administration, as determined by Western blotting. The data are expressed as mean ± SEM. ^∗^*P* < 0.05 compared with the control group and ^#^*P* < 0.05 compared with the hyperoxaluria group.

Expression of Ang II and AT1R was detected *in vitro* and *in vivo* to evaluate the activation of RAS. NRK-52E cells showed upregulation of AT1R after exposure to COM at both mRNA (Figure 2(a)) and protein (Figure 2(b)) levels. The serum concentration of Ang II was also increased in the hyperoxaluria group compared with the untreated control group (Figure 2(c)). Western blotting examination of experimental rat kidneys indicated upregulation of AT1R expression in the hyperoxaluria group compared with the untreated control group (Figure 2(d)).

### 3.4. ROS and NADPH Oxidase Were Upregulated under Stimulation of CaOx Crystals

High concentrations of Ang II had been proven to increase ROS production [9, 10]. Apocynin was used as an inhibitor of NADPH oxidase. Expression of Nox2 and Nox4 was detected to reflect the activity of NADPH oxidase. Activities of SOD and CAT and levels of MDA and 8-OHdG were measured to evaluate the intracellular oxidative stress.

**Figure 3:**
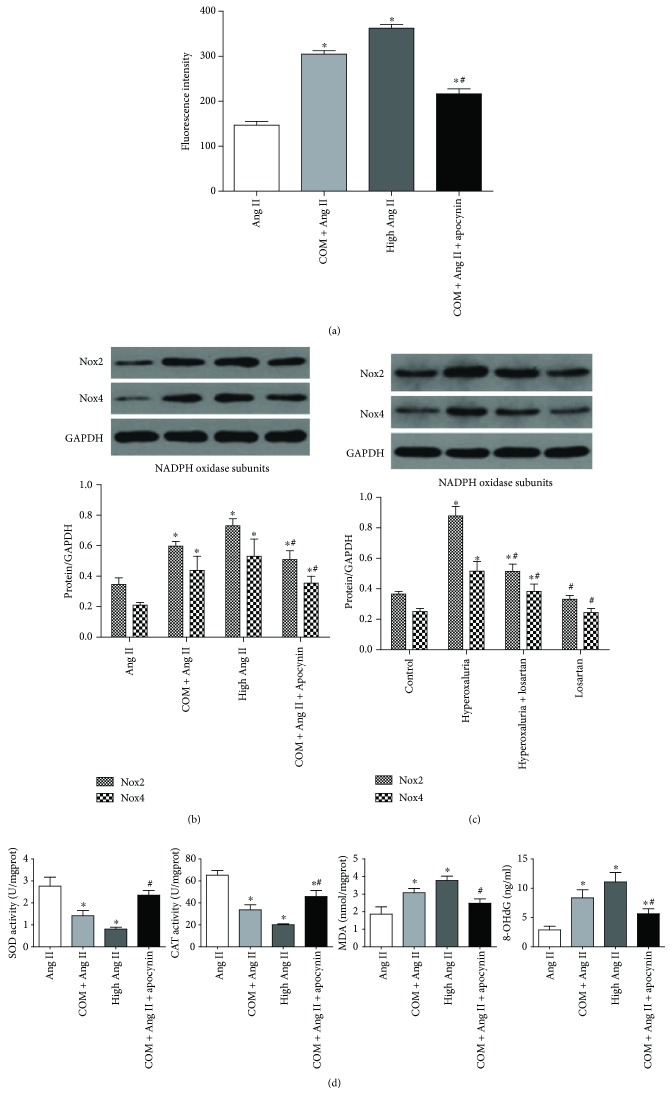
ROS and NADPH oxidase were upregulated under stimulation of CaOx crystals. (a) NRK-52E cells were induced by COM (1 mM) for 6 h with or without apocynin preincubation, and then intracellular ROS was detected by flow cytometry. Expression of Nox2 and Nox4 was upregulated in CaOx-induced NRK-52E cells (b) and rat kidneys in the hyperoxaluria group (c), as determined by Western blotting analysis, and the alteration could be reversed by apocynin or losartan administration. (d) COM (1 mM) reduced cellular SOD and CAT activities and increased MDA and 8-OHdG expression in NRK-52E cells which was reversed by apocynin administration. The data are expressed as mean ± SEM. ^∗^*P* < 0.05 compared with the Ang II group or control group and ^#^*P* < 0.05 compared with the COM + Ang II group or hyperoxaluria group.

The results showed that COM markedly increased intracellular ROS production in accordance with the high concentration of the Ang II group (Figure 3(a)). Expression of NADPH oxidase subunits (Nox2 and Nox4) was upregulated in both CaOx-induced NRK-52E cells and rat kidneys in the hyperoxaluria group by Western blotting analysis (Figures 3(b) and 3(c)). In addition, COM reduced cellular SOD and CAT activities and increased MDA and 8-OHdG levels, prompting a state of oxidative stress (Figure 3(d)). In contrast, preincubation of NRK-52E cells with apocynin reversed the ROS overproduction and activation of NADPH oxidase induced by COM (Figures 3(a)–3(d)). These results suggested that the activity of NADPH oxidase was enhanced by CaOx crystals and might be involved in the CaOx-induced ROS overproduction and oxidative stress.

### 3.5. CaOx Activated the ROS-Mediated Nuclear Factor-*κ*B (NF-*κ*B) Pathway and Expression of Stone-Related Proteins

**Figure 4:**
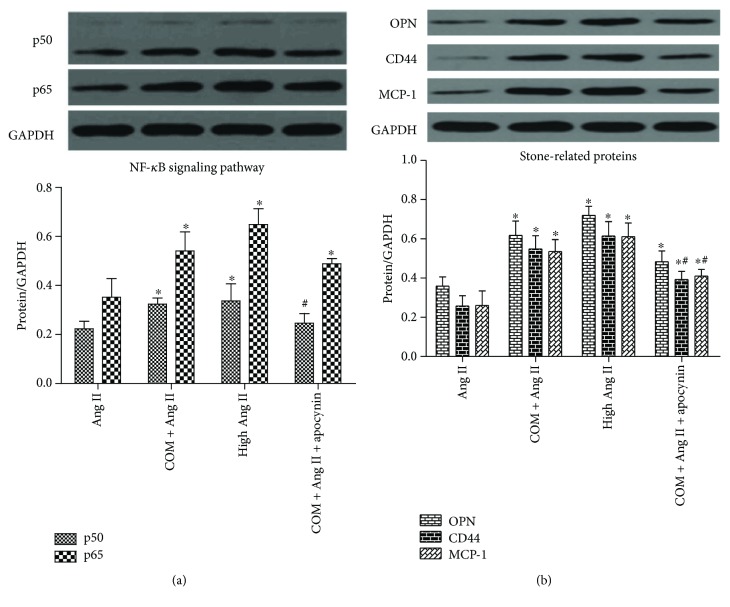
CaOx activated the ROS-mediated NF-*κ*B pathway and expression of stone-related proteins. NRK-52E cells were induced by COM (1 mM) for 6 h with or without apocynin preincubation, and then NF-*κ*B subunits (p50 and p65) (a) and expression of stone-related proteins (OPN, CD44, and MCP-1) (b) were detected by Western blotting. The data are expressed as mean ± SEM. ^∗^*P* < 0.05 compared with the Ang II group and ^#^*P* < 0.05 compared with the COM + Ang II group. ^∗#^*P* < 0.05 compared with the Ang II group and the COM + Ang II group.

The NF-*κ*B pathway is involved in the production of many stone-related proteins in response to oxidative stress [11, 12]. We examined NF-*κ*B pathway activity and expression of stone-related proteins (OPN, CD44, and MCP-1) in response to CaOx treatment. The results of Western blotting analysis showed that treatment with CaOx crystals significantly increased protein expression of NF-*κ*B subunits (p50 and p65) (Figure 4(a)) and stone-related proteins (OPN, CD44, and MCP-1) (Figure 4(b)). Apocynin partially reversed the increased NF-*κ*B pathway activity and expression of stone-related proteins (Figures 4(a) and 4(b)).

### 3.6. CaOx-Induced ROS Generation and Overproduction of Stone-Related Proteins Were Activated via Ang II/AT1R

**Figure 5:**
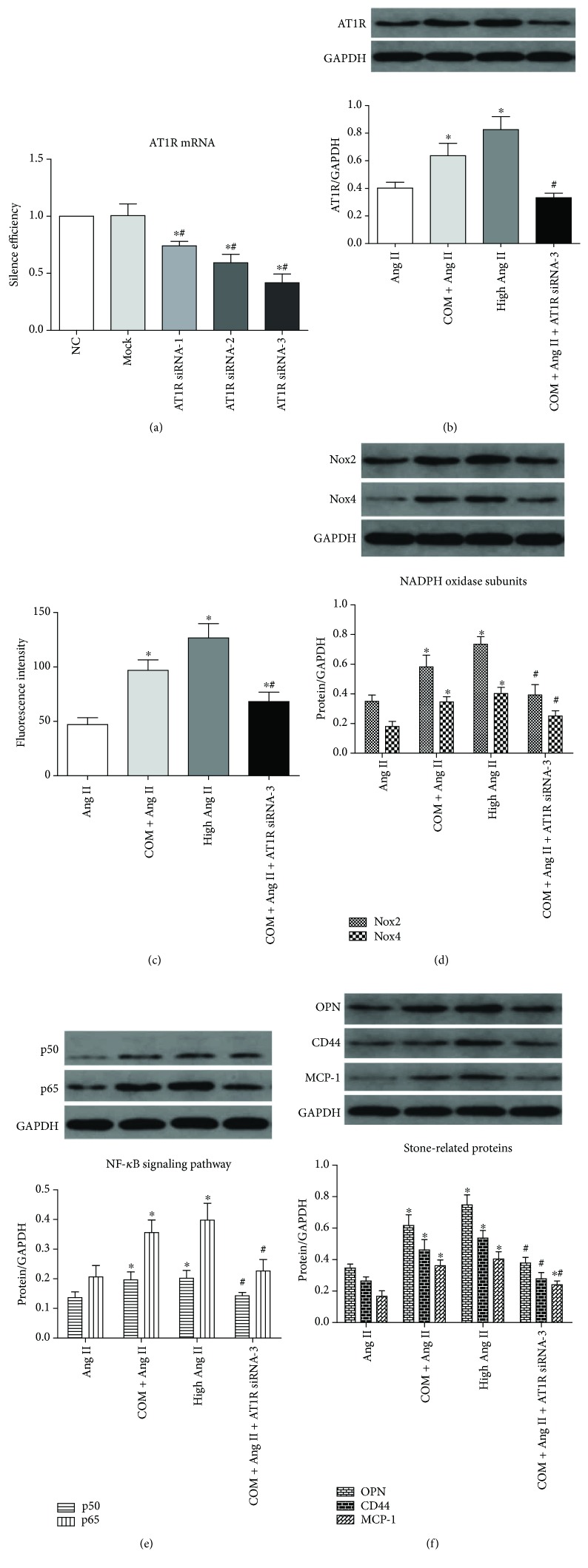
CaOx induced ROS generation and overproduction of stone-related proteins was activated via Ang II/AT1R. (a) The mRNA level of AT1R in NRK-52E cells transfected with AT1R siRNA-1, siRNA-2, or siRNA-3 compared with the negative control group (NC group) (scrambled siRNA transfected) and the mock group (transfection reagent treated only). ^∗^*P* < 0.05 compared with the NC group and ^#^*P* < 0.05 compared with the mock group. (b) The protein level of AT1R was downregulated in the COM + Ang II + AT1R siRNA-3 group compared with the COM + Ang II group. (c) The ROS generation was decreased in the COM + Ang II + AT1R siRNA-3 group compared with the COM + Ang II group. The expression of NADPH oxidase subunits (Nox2 and Nox4) (d), NF-*κ*B subunits (p50 and p65) (e), and stone-related proteins (OPN, CD44, and MCP-1) (f) in the COM + Ang II and COM + Ang II + AT1R siRNA-3 groups was detected by Western blotting in NRK-52E cells. The data are expressed as mean ± SEM. ^∗^*P* < 0.05 compared with the Ang II group and ^#^*P* < 0.05 compared with the COM + Ang II group. ^∗#^*P* < 0.05 compared with the Ang II group and the COM + Ang II group.

In the above results, we showed that both Ang II/AT1R and ROS levels were increased by COM treatment and high Ang II promoted ROS generation. Next, we tried to determine whether CaOx-induced ROS overproduction was activated via Ang II/AT1R. The mRNA levels of AT1R in NRK-52E cells transfected with AT1R siRNA-1, siRNA-2, or siRNA-3 were decreased compared with the negative control group (NC group, scrambled siRNA transfected) and the mock group (transfection reagent treated only) (Figure 5(a)). The AT1R siRNA-3-transfected group achieved the best silencing and was used in subsequent experiments. The protein level of AT1R was downregulated in the COM + Ang II + AT1R siRNA-3 group compared with the COM + Ang II group, which validated the silencing effect of the siRNA (Figure 5(b)). These results showed that downregulation of AT1R remarkably inhibited the activation of ROS (Figure 5(c)) and expression of NADPH oxidase subunits (Nox2 and Nox4) (Figure 5(d)). Furthermore, expression of NF-*κ*B subunits (p50 and p65) (Figure 5(e)) and stone-related proteins (OPN, CD44, and MCP-1) (Figure 5(f)) was decreased in the AT1R siRNA-3-transfected group.

### 3.7. Losartan Downregulated NF-*κ*B Pathway Activity and Stone-Related Protein Expression by Attenuation of Renal Tubular Cell Oxidative Stress

**Figure 6:**
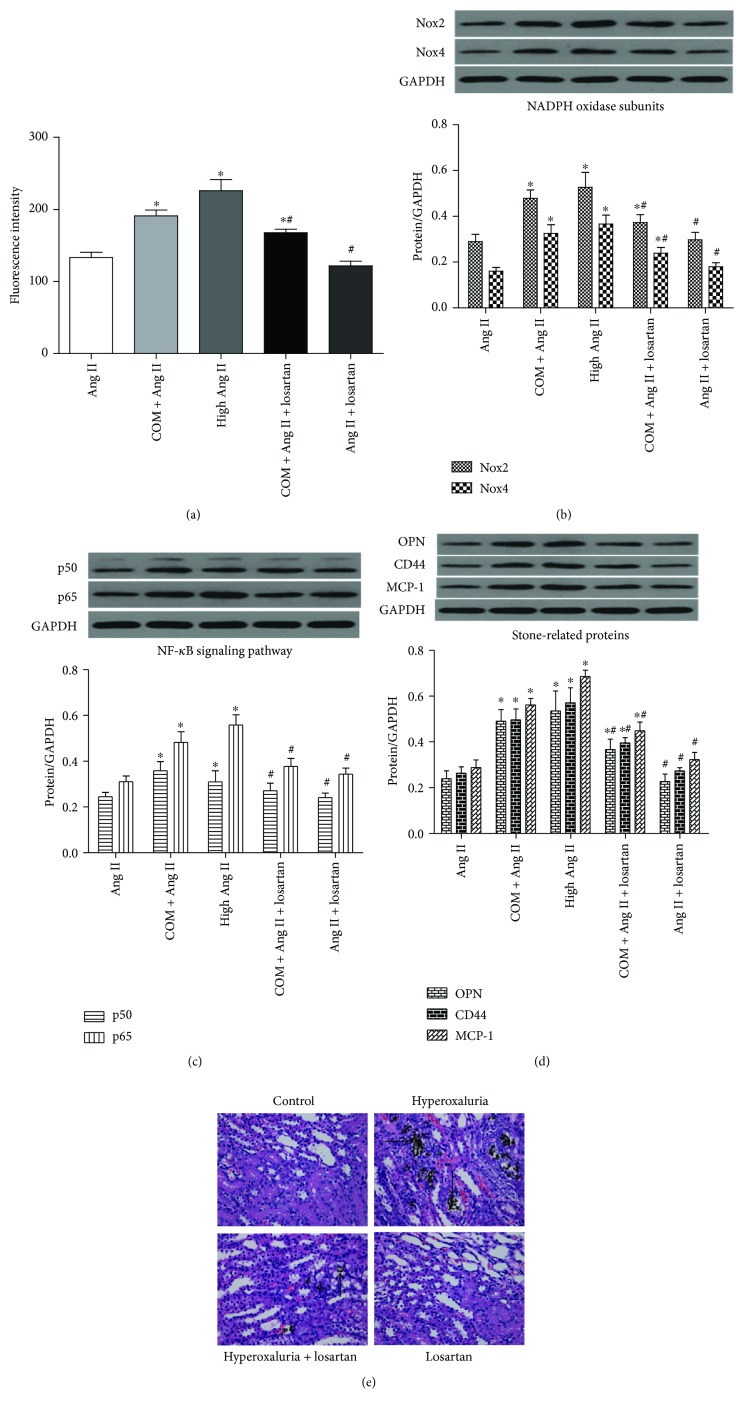
Losartan downregulated NF-*κ*B pathway activity and stone-related protein expression by attenuation of renal tubular cell oxidative stress. (a) NRK-52E cells were induced by COM (1 mM) for 6 h with or without losartan preincubation, and then intracellular ROS was detected by flow cytometry. The expression of NADPH oxidase subunits (Nox2 and Nox4) (b), NF-*κ*B subunits (p50 and p65) (c), and stone-related proteins (OPN, CD44, and MCP-1) (d) was decreased in the COM + Ang II + losartan group compared with the COM + Ang II group in NRK-52E cells. (e) Losartan administration ameliorated the CaOx depositions (black arrow) compared with the rat kidneys of the hyperoxaluria group (Von Kossa staining, magnification: ×400). The data are expressed as mean ± SEM. ^∗^*P* < 0.05 compared with the Ang II group and ^#^*P* < 0.05 compared with the COM + Ang II group. ^∗#^*P* < 0.05 compared with the Ang II group and the COM + Ang II group.

To elucidate the effect of losartan on NF-*κ*B pathway activity and stone-related protein expression in renal tubular cells as well as its association with ROS production, NRK-52E cells were preincubated with losartan for 1 h and then treated with COM for 6 h. As shown in Figure 6, losartan attenuated ROS production (Figure 6(a)) and NADPH oxidase activity (Figure 6(b)) in the presence of COM, meanwhile the NF-*κ*B pathway activity (p50 and p65) (Figure 6(c)) and expression of stone-related proteins (OPN, CD44, and MCP-1) (Figure 6(d)) were decreased at protein levels. As a proof, renal crystal depositions were also significantly ameliorated in rat kidneys of the losartan group compared with the hyperoxaluria group, as examined by Von Kossa staining (Figure 6(e)).

## 4. Discussion

Over the past decades, mechanisms in regard to formation of kidney stones are poorly understood. CaOx is the major mineral constituent of most kidney stones. The formation of CaOx stones involves crystal nucleation, growth, aggregation, and finally retention within the kidneys. Many hypotheses such as Randall's plaques or plugs theory, renal tubular cell inflammatory injury theory, were proposed to explain the origin and formation of kidney stones [13, 14]. Crystal-cell interaction is thought as one of the earliest processes in the formation of urinary stone disease [15]. Exposure to high levels of oxalate or CaOx crystals results in excess production of cellular ROS, followed by inflammation and injury of renal tubular cells [2]. Increasing evidence indicates that ROS is involved in the formation of CaOx stones by regulating multiple signaling pathways including NF-*κ*B and mitogen-activated protein kinase (MAPK) [16–18]. Activation of these transduction pathways leads to elevation of many stone-related proteins such as OPN, CD44, MCP-1, and Tamm-Horsfall protein, which affects cell-crystal interactions and stone formation [3].

NADPH oxidase is one of the major sources of ROS in response to oxalate or CaOx crystals [14]. According to the Nox catalytic subunit, NADPH oxidases are divided into seven isoforms consisting of Nox1–5, Duox1, and Duox2. Among these Nox isoforms, Nox4 and Nox2 are abundantly expressed in the kidney and have attracted broad investigations [4, 19, 20]. Apocynin, a common NADPH oxidase inhibitor, reverses renal injury and significantly decreases kidney deposition of CaOx crystals in a rat model of hydroxyproline-induced hyperoxaluria [21].

The RAS regulates homeostasis of electrolytes, body fluids, and blood pressure under physiological conditions. It has been recognized as a key regulator of many cardiovascular diseases such as hypertension, myocardial ischemia, and congestive heart failure [22–24]. High levels of Ang II were reported to increase ROS production in cardiomyocytes [25]. Recent studies showed activation of the RAS in a hyperoxaluric rat model, which might be associated with ROS production and CaOx crystallization [16, 26]. Decreasing angiotensin production or blocking angiotensin receptors may reduce OPN expression and ameliorate crystal deposition [8]. These previous studies implied that Ang II might have a regulating effect on ROS production and CaOx stone formation.

In our study, we explored the physiological concentration of Ang II, which had little impact on ROS generation. The results showed that 1 nM Ang II was able to simulate the physiological state and was adopted as a physiological concentration in subsequent experiments. Ang II at 1 *μ*M significantly increased ROS generation and was used as a positive control. Next, we detected the expression of Ang II/AT1R in COM-treated NRK-52E cells and in hyperoxaluric rats. The results showed that the serum Ang II concentration was increased in hyperoxaluric rats and AT1R expression was increased in kidney tissues and the cell line. We also examined ROS production and OS status after treatment with CaOx *in vivo* and *in vitro*. The results confirmed that intracellular ROS production and lipid peroxidation levels were increased by CaOx treatment. The activity of NADPH oxidase was enhanced by upregulation of Nox2 and Nox4 under CaOx exposure, and apocynin reversed this increased effect. We also found that the activity of NF-*κ*B and expression of stone-related proteins (OPN, CD44, and MCP-1) were increased by CaOx treatment and similarly reversed by apocynin.

Furthermore, we examined the relationship between Ang II/AT1R and ROS production. Our *in vivo* results demonstrated that CaOx-induced ROS upregulation was mediated by Ang II/AT1R. Moreover, inhibition of AT1R by losartan or AT1R siRNA attenuated ROS production and oxidative stress levels in renal tubular cells by inhibiting NADPH oxidase. The NF-*κ*B pathway activity and expression of stone-related proteins were downregulated simultaneously.

There are some limitations in our study. First, we merely investigated total ROS production and expression of NADPH oxidase subunits, but did not refer to ROS generated by mitochondria. Second, the role of AT2R under stimulation by CaOx was not involved in this study. Moreover, the detailed mechanism of Ang II/AT1R-mediated expression of NADPH oxidase and production of ROS is yet to be studied.

## 5. Conclusion

In summary, the present study provided evidence of upregulated Ang II/AT1R in NRK-52E cells treated with CaOx and in hyperoxaluric rat kidneys. We preliminarily demonstrated that CaOx-induced ROS and stone-related protein upregulation were mediated by Ang II/AT1R via activation of NADPH oxidase. Losartan reduced renal tubular cell expression of stone-related proteins and renal crystallization via inhibiting NADPH oxidase and oxidative stress. These findings indicate that AT1R inhibitor losartan might be a promising preventive and therapeutic candidate for hyperoxaluria nephrolithiasis.

## Figures and Tables

**Table 1:** Sequences of the siRNAs.

siRNA	Forward	Reverse
AT1R siRNA-1	GCGUCUUUCUUCUCAAUCUTT	AGAUUGAGAAGAAAGACGCTT
AT1R siRNA-2	GCCAGUGUGUUCCUUCUCATT	UGAGAAGGAACACACUGGCTT
AT1R siRNA-3	CAGCUGUCAUCCACCGAAATT	UUUCGGUGGAUGACAGCUGTT
Scrambled siRNA	UUCUCCGAACGUGUCACGUTT	ACGUGACACGUUCGGAGAATT

## References

[1] Neisius A., Preminger G. M. (2013). Stones in 2012: epidemiology, prevention and redefining therapeutic standards. *Nature Reviews Urology*.

[2] Zhang J., Wang Q., Xu C. (2017). MitoTEMPO prevents oxalate induced injury in NRK-52E cells via inhibiting mitochondrial dysfunction and modulating oxidative stress. *Oxidative Medicine and Cellular Longevity*.

[3] Khan S. R. (2013). Reactive oxygen species as the molecular modulators of calcium oxalate kidney stone formation: evidence from clinical and experimental investigations. *The Journal of Urology*.

[4] Sedeek M., Nasrallah R., Touyz R. M., Hébert R. L. (2013). NADPH oxidases, reactive oxygen species, and the kidney: friend and foe. *Journal of the American Society of Nephrology*.

[5] Joshi S., Peck A. B., Khan S. R. (2013). NADPH oxidase as a therapeutic target for oxalate induced injury in kidneys. *Oxidative Medicine and Cellular Longevity*.

[6] Joshi S., Saylor B. T., Wang W., Peck A. B., Khan S. R. (2012). Apocynin-treatment reverses hyperoxaluria induced changes in NADPH oxidase system expression in rat kidneys: a transcriptional study. *PLoS One*.

[7] Tsuji H., Wang W., Sunil J. (2016). Involvement of renin–angiotensin–aldosterone system in calcium oxalate crystal induced activation of NADPH oxidase and renal cell injury. *World Journal of Urology*.

[8] Umekawa T., Hatanaka Y., Kurita T., Khan S. R. (2004). Effect of angiotensin II receptor blockage on osteopontin expression and calcium oxalate crystal deposition in rat kidneys. *Journal of the American Society of Nephrology*.

[9] Cabello-Verrugio C., Acuña M. J., Morales M. G., Becerra A., Simon F., Brandan E. (2011). Fibrotic response induced by angiotensin-II requires NAD(P)H oxidase-induced reactive oxygen species (ROS) in skeletal muscle cells. *Biochemical and Biophysical Research Communications*.

[10] Su Q., Huo C.-J., Li H.-B. (2017). Renin-angiotensin system acting on reactive oxygen species in paraventricular nucleus induces sympathetic activation *via* AT1R/PKC*γ*/Rac1 pathway in salt-induced hypertension. *Scientific Reports*.

[11] Tozawa K., Yasui T., Okada A. (2008). NF–*κ*B activation in renal tubular epithelial cells by oxalate stimulation. *International Journal of Urology*.

[12] Lu Y., Qin B., Hu H. (2016). Integrative microRNA-gene expression network analysis in genetic hypercalciuric stone-forming rat kidney. *PeerJ*.

[13] Khan S. R., Canales B. K. (2015). Unified theory on the pathogenesis of Randall’s plaques and plugs. *Urolithiasis*.

[14] Khan S. R. (2014). Reactive oxygen species, inflammation and calcium oxalate nephrolithiasis. *Translational Andrology and Urology*.

[15] Aggarwal K. P., Narula S., Kakkar M., Tandon C. (2013). Nephrolithiasis: molecular mechanism of renal stone formation and the critical role played by modulators. *BioMed Research International*.

[16] Toblli J. E., Cao G., Casas G., Stella I., Inserra F., Angerosa M. (2005). NF-*κ*B and chemokine-cytokine expression in renal tubulointerstitium in experimental hyperoxaluria. Role of the renin-angiotensin system. *Urological Research*.

[17] Koul H. K., Menon M., Chaturvedi L. S. (2002). COM crystals activate the p38 mitogen-activated protein kinase signal transduction pathway in renal epithelial cells. *Journal of Biological Chemistry*.

[18] Chaturvedi L. S., Koul S., Sekhon A., Bhandari A., Menon M., Koul H. K. (2002). Oxalate selectively activates p38 mitogen-activated protein kinase and c-Jun N-terminal kinase signal transduction pathways in renal epithelial cells. *Journal of Biological Chemistry*.

[19] Holterman C. E., Read N. C., Kennedy C. R. J. (2015). Nox and renal disease. *Clinical Science*.

[20] Thallas-Bonke V., Jandeleit-Dahm K. A. M., Cooper M. E. (2015). Nox-4 and progressive kidney disease. *Current Opinion in Nephrology and Hypertension*.

[21] Zuo J., Khan A., Glenton P. A., Khan S. R. (2011). Effect of NADPH oxidase inhibition on the expression of kidney injury molecule and calcium oxalate crystal deposition in hydroxy-L-proline-induced hyperoxaluria in the male Sprague–Dawley rats. *Nephrology Dialysis Transplantation*.

[22] Te Riet L., van Esch J. H. M., Roks A. J. M., van den Meiracker A. H., Danser A. H. J. (2015). Hypertension: renin–angiotensin–aldosterone system alterations. *Circulation Research*.

[23] Schmieder R. E., Hilgers K. F., Schlaich M. P., Schmidt B. M. W. (2007). Renin-angiotensin system and cardiovascular risk. *The Lancet*.

[24] Ferreira A. J., Santos R. A. S., Bradford C. N. (2010). Therapeutic implications of the vasoprotective axis of the renin-angiotensin system in cardiovascular diseases. *Hypertension*.

[25] Chiu C.-Z., Wang B.-W., Shyu K.-G. (2014). Angiotensin II and the JNK pathway mediate urotensin II expression in response to hypoxia in rat cardiomyocytes. *Journal of Endocrinology*.

[26] Yoshioka I., Tsujihata M., Akanae W., Nonomura N., Okuyama A. (2011). Angiotensin type-1 receptor blocker candesartan inhibits calcium oxalate crystal deposition in ethylene glycol-treated rat kidneys. *Urology*.

